# Pancreaticojejunostomy with or without reinforcement after pancreaticoduodenectomy: surgical technique of ligamentum teres hepatis wrap around pancreaticojejunostomy

**DOI:** 10.1186/s12957-018-1484-5

**Published:** 2018-09-07

**Authors:** Maurizio Zizzo, Lara Ugoletti, Andrea Morini, Antonio Manenti, Filippo Lococo, Claudio Pedrazzoli

**Affiliations:** 1 Department of Oncology and Advanced Technologies, Surgical Oncology Unit, Azienda USL-IRCCS di Reggio Emilia, 42123 Reggio Emilia, Italy; 2 Clinical and Experimental Medicine PhD Program, University of Modena and Reggio Emilia, Modena, Italy; 3 Department of General Surgery, University of Modena and Reggio Emilia – Polyclinic, 41124 Modena, Italy; 4 Department of Oncology and Advanced Technologies, Thoracic Surgery Unit, Azienda USL-IRCCS di Reggio Emilia, 42123 Reggio Emilia, Italy

**Keywords:** Ligamentum teres hepatis, Postoperative pancreatic fistula, Pancreaticoduodenectomy, Pancreatic cancer

## Abstract

In a previous issue of the Journal, Zhong et al. reported a retrospective study that compared the perioperative outcomes of the mesh-reinforced pancreaticojejunostomy with conventional pancreaticojejunostomy. They concluded that mesh-reinforced pancreaticojejunostomy was a safe and effective technique, as it provided a safe anchor site for suture, thus reducing the risk of pancreatic leakage. Considering these encouraging results, we present a further simple technique using ligamentum teres hepatis wrap around pancreatojejunostomy for prevention of postoperative pancreatic fistula after pancreaticoduodenectomy.

Dear Editor,

We really appreciated the paper written by Zhong et al. entitled “Mesh-reinforced pancreaticojejunostomy versus conventional pancreaticojejunostomy after pancreaticoduodenectomy: a retrospective study of 126 patients”, and published in the *World Journal of Surgical Oncology* in March 2018 [1]. The authors retrospectively analyzed 126 patients who underwent pancreaticoduodenectomy for pancreatic cancer with the aim of comparing the perioperative outcomes of the mesh-reinforced pancreaticojejunostomy (65 patients) with conventional pancreaticojejunostomy (61 patients) [1]. Mesh-reinforced pancreaticojejunostomy turned out as a safe and effective technique, as it provided: (i) a safe anchor site for suture to avoid anastomotic laceration, especially in soft and fragile pancreatic remnant textures, and postoperative bleeding; (ii) a compression to pancreatic tissue that minimized the chance of pancreatic leakage and bleeding; and (iii) a stimulation of fibroblast growth and of anastomotic healing process [1].

Another conclusion that we considered of considerable importance was the absence of statistically significant differences between the two groups in terms of intra-abdominal infection, despite the implantation of a foreign body [1].

More than 80 different surgical methods have been described in order to perform a safe and effective pancreaticojejunostomy, but none of them has been proven to be superior to the others [1].

Considering the encouraging results presented by Zhong et al. [1], we would like to suggest a further simple technique using ligamentum teres hepatis wrap around pancreaticojejunostomy we have been using at our Center since June 2017. It does not require any heterologous material. In resectable pancreatic head cancer patients (based on multidisciplinary assessment and National Comprehensive Cancer Network guidelines), we performed a pylorus-preserving pancreaticoduodenectomy.

Steps of surgical procedures were the following ones: (i) a transverse subcostal abdominal incision was always performed; (ii) if the falciform ligament was not previously dissected/resected, the pedicled ligamentum teres hepatis was mobilized after division of the round ligament close to the umbilicus; (iii) the falciform ligament was always dissected from the ventral abdominal wall up to the junction with the coronary ligament; (iv) first, a Kocher maneuver was performed to assess relationship between the tumor and the superior mesenteric artery; (v) the second maneuver performed to assess resectability developed a plane of dissection between the anterior surface of the superior mesenteric vein/portal vein confluence and posterior surface of pancreatic neck to exclude tumor involvement; (vi) if resectability was confirmed, “demolition” phase of pancreaticoduodenectomy was completed; (vii) pancreaticojejunostomy with end-to-side anastomosis was always performed in a single layer of interrupted 4-0 polydioxanone suture (in all cases, we placed an external drainage in the remaining main pancreatic duct); (viii) hepatico-jejunal and duodeno-jejunal anastomoses were always performed in a single layer of interrupted 4–0 and 3-0 polydioxanone suture, respectively.

Technical innovation consisted of many following steps: (i) ligamentum teres hepatis (adequately mobilized when starting surgical procedure) was passed under pancreaticojejunostomy until covering posterior wall of the anastomosis; (ii) we completely wrapped the anastomosis with ligamentum teres hepatis; and (iii) subsequently, we anchored the ligamentum teres hepatis to the proximal side of the same, to the pancreatic capsule, and to the seromuscolar layer of the jejunal loop with interrupted 5-0 polydioxanone sutures, along the two angles and anterior wall of the anastomosis. A representation of the final result is shown in Fig. 1.

**Fig. 1:**
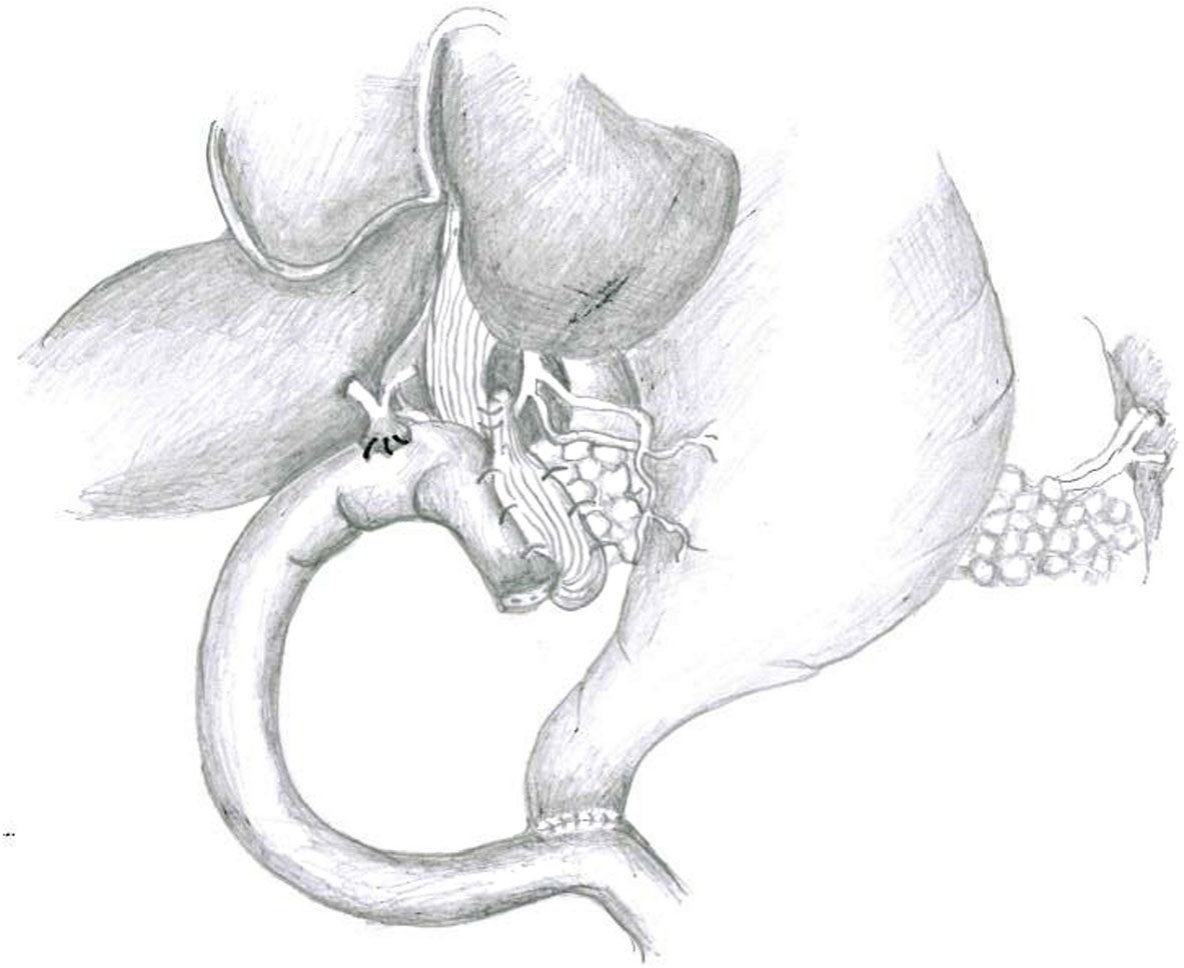
Ligamentum teres hepatis wrap around pancreaticojejunostomy

At present, ligamentum teres hepatis and falciform ligament are successfully used to prevent postoperative pancreatic fistulas following distal pancreatectomy [2] and erosion bleeding from gastroduodenal artery stump following pancreaticoduodenectomy [3]. This promising surgical procedure is routinely used in some Asian centers, while it is uncommon in Europe and the USA [4]. Nevertheless, no case study reported use of ligamentum teres hepatis to prevent pancreatic leakage following pancreaticoduodenectomy.

Starting in June 2017, we successfully employed this new technique in 14 cases. According to latest guidelines by International Study Group of Pancreatic Surgery (ISGPS) [5], just two biochemical leaks (BLs) were reported. No other critical complications were observed. However, our results are very preliminary and it is currently not possible to reach definitive conclusions about the efficacy of this new technique in the reduction of morbidity related to pancreaticoduodenectomy (rate of postoperative pancreatic fistula in particular).

Therefore, we stress the need for a case-control study, cohort study, or prospective randomized study which might confirm the technique’s effectiveness, as it happened for the new technique by Zhong et al. [1].

## Abbreviations

BL: Biochemical leak
ISGPS: International Study Group of Pancreatic Surgery
